# Prediction of cognitive impairment using higher order item response theory and machine learning models

**DOI:** 10.3389/fpsyt.2023.1297952

**Published:** 2024-03-01

**Authors:** Lihua Yao, Yusuke Shono, Cindy Nowinski, Elizabeth M. Dworak, Aaron Kaat, Shirley Chen, Rebecca Lovett, Emily Ho, Laura Curtis, Michael Wolf, Richard Gershon, Julia Yoshino Benavente

**Affiliations:** ^1^Department of Medical Social Sciences, Northwestern University Feinberg School of Medicine, Chicago, IL, United States; ^2^School of Community and Global Health, Claremont Graduate University, Claremont, CA, United States; ^3^Transitional Year Residency, Aurora St. Luke's Medical Center, Milwaukee, WI, United States

**Keywords:** MyCog, NIH Toolbox, machine learning, deep learning, IRT, higher order item response theory, impairment, cognitive impairment

## Abstract

Timely detection of cognitive impairment (CI) is critical for the wellbeing of elderly individuals. The MyCog assessment employs two validated iPad-based measures from the NIH Toolbox^®^ for Assessment of Neurological and Behavioral Function (NIH Toolbox). These measures assess pivotal cognitive domains: Picture Sequence Memory (PSM) for episodic memory and Dimensional Change Card Sort Test (DCCS) for cognitive flexibility. The study involved 86 patients and explored diverse machine learning models to enhance CI prediction. This encompassed traditional classifiers and neural-network-based methods. After 100 bootstrap replications, the Random Forest model stood out, delivering compelling results: precision at 0.803, recall at 0.758, accuracy at 0.902, F1 at 0.742, and specificity at 0.951. Notably, the model incorporated a composite score derived from a 2-parameter higher order item response theory (HOIRT) model that integrated DCCS and PSM assessments. The study's pivotal finding underscores the inadequacy of relying solely on a fixed composite score cutoff point. Instead, it advocates for machine learning models that incorporate HOIRT-derived scores and encompass relevant features such as age. Such an approach promises more effective predictive models for CI, thus advancing early detection and intervention among the elderly.

## 1 Introduction

Cognitive impairment (CI) is known to be a significant public health concern; it has profound impact on older adults. Oftentimes, mild CI (MCI) goes undetected, and clinical detection. Clinicians may solely rely on patients proactively self-reporting concerns when only a third typically acknowledge any cognitive problems (1–4).

Early detection of CI has many potential benefits for patients and their families. Early detection may reveal reversible or treatable causes (e.g., depression and vitamin B12 deficiency) (Office of Disease Prevention and Health Promotion, n.d.). When CI is present, early detection may allow patients and families time to emotionally adjust and plan for the future, opportunities for treatments to reduce symptoms, (optimizing functional independence and quality of life), and the ability to address/prevent safety concerns (e.g., driving and home environment) (5). Our goal was to develop a brief measure (< 10 minutes) for use in primary care settings for the early detection of CI including dementia (CID). The MyCog assessment uses adapted versions of two well-validated iPad-based measures from the NIH Toolbox^®^ for Assessment of Neurological and Behavioral Function Cognitive Battery (NIHTB-CB) that address two of the first domains to show CI: Picture Sequence Memory (PSM) which assesses episodic memory and Dimensional Change Card Sort Test (DCCS) measuring cognitive flexibility (6–8). The purpose of this study was to pilot, refine, and preliminarily validate the MyCog Assessment to detect CID in a sample of community dwelling older adults in primary care. In total, 86 participants were recruited from an existing cohort study and completed a brief, in-person interview. CI was determined based on a diagnosis of dementia or CI in their medical record or based on a comprehensive cognitive battery performed within the past 18 months. In addition to MyCog DCCS and PSM assessments, participants also took three external assessments from the Mobilke Toolbox: Arrow Matching (ARW), Sequences (MFS), and Number-Symbol Match (NSM). Mobile Toolbox assessments are self-administered on smartphones. The five assessments will be explained in more detail in the next section.

The objective of this study is to identify the optimal derived scores that can distinguish individuals with and without cognitive impairment. The study employs a systematic investigation that evaluates multiple scoring methods within the same study to identify the most effective predictors for accurately classifying individuals into their respective clinical groups. Specifically, the study focuses on defining the two-content area scores and compare it with five-content area scores. The aim is to gain a deeper understanding of the underlying scoring of the subtests and to test the final composite “MyCog” score for its ability to differentiate between groups. In addition, the study employs machine learning (ML) models to enhance prediction accuracy. Non-parametric models are used by ML to improve model accuracy by fitting and representing the data in a more flexible manner (9). The study investigates the most important variables or "features" collected by the MyCog assessment that can predict cognitive status.

Overall, the study aims to provide a comprehensive analysis of different assessments and the different scoring methods, and machine learning techniques that can effectively distinguish between individuals with and without cognitive impairment, with less assessments. We conducted a study involving 86 clinical patients who underwent NIH Toolbox assessments, PSM and DCCS, administered via iPad. Additionally, patients self-administered Mobile Toolbox assessments, specifically ARW, MFS, and NSM using their smartphones. Different models for the composite score and different machine learning models with different feature spaces were examined for the purpose of better prediction of MCI.

## 2 Methods

### 2.1 Real data

In this study, we used data from patients who took the five assessments: MyCog DCCS and PSM, and Mobile Toolbox ARW, MFS, and NSM. After cleaning and merging, there were 86 patients with their five cognitive assessment data information that contains their item responses for the five content area tests and responses times for three tests: ARW, PSM, and DCCS. Each patient also has demographic information for age, race, education, and income. Their impairment status is categorized as 0/1 with 0 indicating normal cognition and 1 indicating CI. Table 1 shows the demographic and clinical information for the 86 patients. Cognitive status is also broken out by gender. For example, out of 60 female patients, there were 46 normal cognition and 14 with CI. For the education level, Master's degree, represented by 9, had the most percentage 32 followed by 23 percentage of Bachelor's Degree, represented by 8. Table 2 show the statistics of response times (in s) for the three content area ARW, DCCS, and PSM for the 86 patients.

**Table 1:** Statistics of the 86 patients.

**Category**	**Numerical value**	**Data set value**	**Count**	**Percent**
	**After scaling**			
Impaired	0	Normal Cognition	67	78
	1	Cognitive Impairment	19	22
Gender	0	F	60(46N,14I)	70
	1	M	26(21N,5I)	30
Race	0	Asian	4	5
	1	Black	11	13
	2	Unreported	1	1
	3	White	70	81
Income	0	2	2	2
	1	3	3	3
	2	4	3	3
	3	5	8	9
	4	6	10	12
	5	7	12	14
	6	8	21	24
	7	9	26	30
	8	D	1	1
Education		4	5	6
		6	10	12
		7	3	3
		8	23	27
		9	32	37
		10	5	6
		11	6	7
		777	2	2

**Table 2:** Statistics of the item response times for ARW, DCCS, and PSM (in s).

	**ARW**	**DCCS**	**PSM**
count	86	86	86
mean	47.421	36.049	73
std	11.619	14.909	34
min	31.906	15.301	30
25%	39.207	26.823	52
50%	44.966	33.014	63
75%	52.492	40.248	84
max	94.313	103.959	233

#### 2.1.1 Cognitive measures

##### 2.1.1.1 MyCog DCCS

The MyCog DCCS assesses executive function in general and cognitive flexibility in particular. In this task, two reference visual images that vary by two dimensions—color and shape—are presented and remain in the bottom screen. Respondents are asked to match target visual images that appear individually on the center screen to one of the two reference visual images according to color or shape. The task consists of five blocks of trials: (1) two practice trials for the shape dimension, (2) five (pre-switch) trials for the shape dimension, (3) two practice trials for the color dimension, (4) five (post-switch) trials for the color dimension, and (5) 30 (mixed) trials, in which the target dimension (i.e., color or shape) were switched in a predetermined random order. Responses and response times (RTs) were collected on each trial. RTs were defined as the time elapsed in milliseconds between the onset of the target visual image and the respondent's response. Estimated average testing time is 3 min.

##### 2.1.1.2 MyCog PSM

The MyCog PSM assesses episodic memory. In this task, a series of 12 visual images/pictures depicting independent, non-sequential activities were presented individually on the center screen, along with audio description of the picture content, and then placed into one of twelve box slots. Respondents were instructed to remember the order in which the pictures were presented and in which box each was placed (i.e., encoding). Once all the pictures were presented, they were scrambled to the center of the screen, and the respondents' task was to place the pictures in the boxes in the order of original presentation (i.e., memory recall). Respondents began with a practice trial which is comprised of four picture images, followed by a single test trial with twelve picture images. Estimated average testing time is 5 min.

##### 2.1.1.3 Mobile Toolbox ARW

Mobile Toolbox ARW targets inhibitory control and attention. It is a 50-item test that measures executive attention, which encompasses endogenous attentional processes that are under cognitive control and overlaps considerably with the construct of executive function. Dual task paradigms such as ARW have shown impairments in asymptomatic carriers of familial Alzheimer's disease and individuals with CRCI. This measure requires respondents to indicate the left-right orientation of a centrally presented stimulus while inhibiting attention to potentially incongruent stimuli that surround it. Estimated average testing time is 3 min.

##### 2.1.1.4 Sequences

MFS assesses working memory—ability to recall unrelated information and cognitively manipulate it to produce a new output. MFS is a newly developed measure of working memory with a item bank of 30 different stimuli. Working memory deficits have been previously shown in the context of cognitive decline, particularly with Parkinson's disease and HIV-associated neurocognitive disorders. MFS requires participants to remember a string of letters and numbers and manipulate them in order to put them in an alphabetical and numeric order. Trials begin with strings of three alphanumeric characters and become increasingly challenging, reaching a maximum difficulty of 10 characters. The measure ends when a participant answers three subsequent trials incorrectly. Scores reflect the number of correct trials. Estimated average time is 5 min.

##### 2.1.1.5 Number-symbol match

This measure is administered using a landscape orientation. A key with nine symbols paired with digits (1-9) is presented at the top of the screen. Participants are asked to enter the number associated with the symbol in a row of items below. The primary score is the total number of correct items in 90 seconds. Number-Symbol Match, a 144-item test, indexes several cognitive functions including perception, encoding, and retrieval and the transformation of information stored in active memory and decision-making. Due to the wide range of abilities influencing task performance, this measure provides high sensitivity and low specificity for many types of impairment, including normal age-related decline, dementia, cancer, and multiple sclerosis. Estimated average testing time is 2 min.

#### 2.1.2 Item and test level psychometrics

Regarding internal consistency, Cronbach's alpha values for the five content areas assessed by the tests (DCCS, PSM, ARW, MFS, and NSM) were as follows: 0.866, 0.828, 0.593, 0.833, and 1, respectively. Notably, the first three assessments had no missing values, while the percentage of missing responses for MFS and NSM increased as the testing progressed. This increase in missing responses for MFS and NSM may be attributed to patient fatigue during the testing process or due to the stopping rules implemented by these tests. We are most interested in using the two MyCog cognitive measures (DCCS and PSM). Table 3 presents correlations between cognitive test results measures (DCCS and PSM) and demographic information for the sample of 86 patients. The correlations between the scores (number of correct responses) in all five content areas range from 0.2 to 0.6. Table 4 provides a summary of the mean and standard deviation (SD) scores for the number of correct responses in the five content areas, categorized by demographic factors. The statistical summary indicates that there are no significant performance differences based on gender, race, education, or income. However, it is noteworthy that the scores consistently tend to be lower in the impaired group compared to the normal group.

**Table 3:** Relationships between measures and demographics.

	**Impaired**	**Education**	**Race**	**Income**	**Age**	**Gender**	**PSM**	**DCCS**
Impaired	1	-0.087	-0.136	-0.335	0.268	-0.045	-0.334	-0.284
Education	-0.087	1	-0.105	-0.012	0.095	0.069	-0.07	0.088
Race	-0.136	-0.105	1	0.076	0.125	0.132	0.157	-0.014
Income	-0.335	-0.012	0.076	1	-0.132	0.167	0.097	0.109
Age	0.268	0.095	0.125	-0.132	1	0.067	-0.27	-0.174
Gender	-0.045	0.069	0.132	0.167	0.067	1	-0.331	0.065
PSM	-0.334	-0.07	0.157	0.097	-0.27	-0.331	1	0.268
DCCS	-0.284	0.088	-0.014	0.109	-0.174	0.065	0.268	1

**Table 4:** Measure differences(mean (SD)) among demographics.

	**PSM**	**DCC**	**ARW**	**MFS**	**NSM**
**Impaired**					
0	6 (3)	29 (2)	49 (3)	11 (3)	31 (8)
1	4 (3)	27 (5)	45 (10)	6 (4)	21(8)
**Gender**					
Female	7 (3)	28(3)	48 (4)	10 (4)	31(9)
Male	4 (2)	29(3)	48 (8)	10 (4)	25(9)
**Race**					
Asian	5 (3)	27(5)	49 (2)	10 (4)	30(3)
Black	5 (2)	29(2)	47 (5)	10 (5)	29(9)
White	6 (3)	28(3)	48 (6)	10 (4)	29(9)
**Education**					
4	5 (4)	24(6)	46 (3)	6 (4)	21(7)
6	5 (4)	27(5)	44 (13)	8 (5)	2(10)
7	6 (4)	29(1)	50 (1)	11 (2)	31(9)
8	6 (3)	29(1)	48 (4)	10 (4)	29(10)
9	6 (3)	29(3)	48 (4)	12 (3)	31(9)
10	6 (2)	30(0)	50 (1)	10 (4)	31(4)
11	6 (4)	30(1)	50 (0)	10 (1)	32(5)
777	4 (1)	30(0)	48 (1)	12 (2)	28(2)
**Income**					
2	7 (1)	29(1)	49 (1)	8 (4)	30(8)
3	3 (3)	27(2)	35 (25)	6 (5)	1(10)
4	7 (3)	29(1)	43 (9)	10 (1)	26(6)
5	6 (4)	29(2)	48 (3)	9 (4)	27(11)
6	4 (3)	26(6)	48 (2)	9 (5)	30(8)
7	6 (4)	28(4)	49 (2)	11 (4)	29(10)
8	6 (3)	29(2)	48 (3)	11 (3)	30(9)

### 2.2 Study design

The study design involves analyzing real data with all available information to identify the best predictors and models for predicting impairment. The analysis will involve comparing the prediction accuracy of using two cognitive assessments vs. five cognitive assessments. Additionally, the study will investigate whether using only the derived composite scores can accurately predict impairment (see next section). Finally, machine learning models will be used to determine if other information can improve prediction accuracy beyond the cognitive assessment.

An initial unsupervised analysis was performed using the KMeans clustering algorithm to group the data into distinct clusters. The elbow method was utilized to determine the optimal number of clusters, and the resulting clusters were analyzed to identify patterns and relationships between the patients' composite scores, income, age, and clinical impairment status.

The supervised analysis is conducted using clinical labels of impairment to identify the factors that affect prediction accuracy. This analysis involves examining the relationships between the features and labels to determine the most relevant variables for predicting clinical impairment. The results of this analysis can help identify potential risk factors and develop diagnostic tools for early detection and intervention.

### 2.3 The item response theory models

There were five distinct areas of content, each with varying numbers of item responses for DCCS, PSM, ARW, MFS, and NSM, with 30, 12, 50, 30, and 144 item responses, respectively. In order to calculate the IRT scores, higher order two-parameter item response theory (HOIRT) was employed (10).

For the combined data across all five content areas, five-dimensional and two-dimensional HOIRT models were utilized using Markov chain Monte Carlo [BMIRT, (11)]. For the five-dimensional HOIRT model (HOIRT-5D), the resulting overall score and scores for the five dimensions for each of the five content areas were represented by SSHO, dccs, psm, arw, mfs, and nsm, respectively. For the two-dimensional HOIRT models (HOIRT-2D), the resulting overall score and scores of the two dimensions for each of the two content areas were represented by SSHO2D, dccs2D, and psm2D.

### 2.4 Composite scores

One way of obtaining composite scores is to run a linear regression for the possible combination of k variables.


(1) $$\begin{array}{l} Impaired=f\left(x_{1},\cdots x_{k}\right), \end{array}$$

where the function *f* is a linear regression function and k is the number of variables applied. Five linear regressions were run from the data and the coefficients are listed in Table 5. The probabilities were derived based on the coefficients in Table 5 and the formula below:


(2) $$\begin{array}{l} P=\sigma \left(f\left(x_{1},\cdots x_{k}\right)\right) \end{array}$$

(3) $$\begin{array}{l} =\frac{exp^{f\left(x_{1},\cdots x_{k}\right)}}{1+exp^{f\left(x_{1},\cdots x_{k}\right)}} \end{array}$$

**Table 5:** Coefficients for the five composite scores.

	**Composite1**	**Composite2**	**Composite3**	**Composite4**	**Composite5**
	**Classical**	**HOIRT5D**	**HOIRT2D**	**HOIRT2D**	**Classical**
Intercept	1.08	-0.07	-0.0066	0.479	-0.129
SSHO		0.0607	2.413	3.137	
arw	0.001	-0.0082			
psm	-0.004	-0.0231	-0.919	-1.165	-0.316
nsm	-0.01	-0.0146			
dccs	-0.16	-0.0279	-1.381	-1.755	-0.115
mfs	-0.03	0.028			
arw-rt		0.000012			
dccs-rt		0.0000009			
psm-rt		-0.00149			
age			0.0068		0.048

*Composite1* is calculated by combining the scores from the five content areas using classical methods. Specifically, the number of correct scores were used for the PSM, MFS, and NSM tests. For the DCCS and ARW tests, accuracy scores were calculated by dividing the number of correct responses by the response time. These accuracy scores were then used to calculate the composite score.

*Composite2* is a composite score that takes into account cognitive ability scores and response time measures for ARW, DCCS, and PSM. It is derived from a linear combination of the six scores and their response times: ssho, dccs, arw, psm, mfs, and nsm, and arw-rt, dccs-rt, and psm-rt^1^. These scores are based on the five-dimensional score estimates from the HOIRT model, which includes both overall score estimates and five domain score estimates using the BMIRT method developed by Yao (11, 12).

*Composite3* is the linear combination of the three scores SSHO2D, dccs2D, and psm2D from the two-dimensional HOIRT model and age.

*Composite4* is the linear combination of the three scores SSHO2D, dccs2D, and psm2D from the two-dimensional HOIRT model.

*Composite5* is the linear combination of the scores from DCCS score accuracy (total score divided by the response time) and number of correct scores for PSM.

### 2.5 Feature space

In this study, various feature spaces were explored to identify the best combination of features for predicting clinical impairment using the data from 86 patients. The feature spaces were designed to investigate the impact of different variables, such as demographics, response time, and cognitive assessments, on the prediction of clinical impairment.

- Feature space *F1* contains all five assessment content areas scores
  {*SSHO, dccs, psm, arw, mfs, nsm*}, three content area response times {*psm*−*rt, arw*−*rt, dccs*−*rt*}^2^, and four demographic information {*race, gender, income, education*} This feature space has 13 features; it is considered as the baseline because it contains the most number of related features.
- Feature space *F2* dropped the four demographic features from *F1* and contains six assessment scores and three response times; it has nine features.
- Feature space *F3* dropped the three response times from F1 and contains 10 features in total {*race, gender, income, education, SSHO, dccs, psm*, *arw, mfs, nsm*}.
- Feature space *F4* has six features and contains the overall score and five content area scores {*SSHO, dccs, psm, arw, mfs, nsm*}.
- Feature space *F5* contains the four demographic information {*race, gender, income, education*}, scores from HOIRT-2D with DCCS and PSM {*SSHO*2*D, dccs*2*D, psm*2*D*}, and two content area response times {*dccs*–*rt, psm*–*rt*}; it has nine features.
- Feature space *F6* dropped the two response times in *F5* and contains seven features in total.
- Feature space *F7* contains scores from HOIRT-2D with DCCS and PSM {*SSHO*2*D, dccs*2*D, psm*2*D*}; it has three features.
- Feature space *F8* has only one feature {*Composite1*}.
- Feature space *F9* has only one feature {*Composite2*}.
- Feature space *F10* has only one feature {*Composite3*}.
- feature space *F11* has two features {*age, Composite4*}.
- Feature space *F12* has only one feature {*Composite5*}.
- Feature space *F13* has three features that used assessment PSM only: {*psm, psm*−*rt, age*}.
- Feature space *F14* has three features that used assessment DCCS only: {*dccs, dccs*−*rt, age*}.
- Feature space *F15* contains two features {*age, SSH*2*D*}.
- Feature space *F16* contains five features {*age, dccs*2*D, psm*2*D*}, and {*dccs*−*rt, psm*−*rt*} .

### 2.6 Machine learning models

The data set of size 86 was split into a training dataset and a testing dataset with a ratio of 80/20. The training and testing datasets had 68 and 18 cases, respectively. After splitting the data, supervised machine learning and deep learning models were applied and compared. For each of the models and model classes, we applied a regular grid search to the training dataset using *GridSearchCV* in *sklearn* (13) with a default 5-fold cross-validation. To limit the risk of data overfitting and bias during model construction, the training dataset are divided into 5 smaller analysis (similar to a training set) and validation (testing) sets. After splitting the training data, 1-fold is used for the validation of the model and developing the aggregated assessment statistics while the other 4-folds are used for building the model. For 5-fold cross-validation, the sample size for each folder/set is approximately 13 or 14. The grid search will evaluate the model on the validation set (one of the five sets) and record the results for each iteration. After all five iterations have completed, the results are aggregated and the best combination of hyperparemeters is selected. Since the quality and quantity of the training data have a significant impact on the accuracy and performance of predictive models, we explored three types of models: hyperplane separation, assembling, and deep learning. Modeling applied from “sklearn” and “Keras” package include as follows: (1) hyperplane separation models (SVM, LRG, Tree, and KNN); (2) assembling models (RF and GB); (3) deep learning models (ANN, FNN, and RNN).

#### 2.6.1 Support vector machine (SVC)

SVC (14) is a supervised machine learning method used for classification and regression. The parameters used is “rbf,” Radial Basis Function, for the kernal and four C values for the strength of the regulation from 1, 10, 50, 100.

#### 2.6.2 Logistic regression (LRG)

LRG (15) is a regression based classification algorithm that takes into account the probability of an outcome. The hyperparameters used for tuning this model include a L2 penalty term, the default solver “lbfgs,” and 20 regularization strength from 0.01 to 5 were implemented.

#### 2.6.3 Decision Tree (Tree)

A supervised learning approach for classification that splits observations based on an optimal cut-point observed in the data based on a varying number of features. Seven numbers from 3 to 30 were chosen for *max*_*depth*.

#### 2.6.4 K-Nearest neighbors (KNN)

A non-parametric algorithm that relies on classifying cases based on their proximity or distance.

#### 2.6.5 Random forest (RF)

RF (16) is a supervised ensemble learning method for classification that combines the results of bootstrapped aggregated independent decision trees. The ensemble is through bootstrapping (17). Ten *n*_*estimators*, or minimum number observations, ranged from 10 to 500.

#### 2.6.6 Gradient boosting (GB)

GB (18) is a class of algorithms for classification that uses tree-based statistical learning techniques. Rather than building ensemble models from multiple independently derived decision trees like RF, the Boosting (18) method is additive and constructs and aggregates decision trees sequentially. We used *GradientBoostingClassifier* from *sklearn*. Three *n*_*estimators* values 10, 30, and 50 were used.

#### 2.6.7 Artificial neural network (ANN)

ANN (19, 20) is a neural network that mimics the way nerve cells work in the human brain. We used *MLPClassifier* from *sklearn*. Cross run of two layer sizes ([(150, 100, 50), (120, 80, 40)]), two slover ([“sgd”, “adam”]), two activation ([“tanh”, “relu”]), two alpha ([0.01, 0.3, 1]), and two learning rate ([“constant”, “adaptive”]) were conducted.

Both tanh and relu are non-linear activation functions^3^.

#### 2.6.8 Feedforward neural network (FNN)

FNN (21) is an artificial neural network, named feedforward neural network. We used *Sequential* imported from *keras*.*models* and five layers with Dense 2000 and activation='relu', which stands for the rectified linear activation unit. The last layer used “sigmoid” as the activation. The difference between this multi-class classification model and the other models is that the output value, which is the scores for each essay, need to be converted or reshaped into a matrix format of binary data; it is called one-hot encoding. That is, if the essay score is 8, then the column 8 of the matrix is 1, and the other columns has value 0. Since the maximum score point for all essays in prompts 1–6 is 12, the matrix has a total of 13 columns.

#### 2.6.9 Recurrent neural network (RNN)

For the RNN process (22), the Keras library was used to create a Sequential model (23) with a single SimpleRNN layer. This type of recurrent neural network is designed to process sequential data and maintain a memory of past inputs. The SimpleRNN layer used in this process has 200 units and uses the hyperbolic tangent activation function (“tanh”). After creating the SimpleRNN layer, three additional Dense layers were added to the model, each with the “relu” activation function. The final layer of the model used the “sigmoid” activation function. During the training process, two epochs were used, with sizes of 64 and 32, respectively. Additionally, a hyperparameter search was conducted using three different batch sizes: 5, 12, and 32.

### 2.7 Evaluation criteria

In machine learning, metrics such as *precision*, *recall* or *sensitivity*, *accuracy*, *F*-score, and *specificity* are typically computed, and they are based on a confusion matrix as shown in Table 6. In the medical field, recall is important; it measures the rate of accurately identify positive/impairment patient. Specificity measures the rate of correctly identifying normal cognition in patients. They are defined below:


(4) $$\begin{array}{l} Precision=\frac{TP}{FP+TP} \end{array}$$

(5) $$\begin{array}{l} Recall=\frac{TP}{FN+TP} \end{array}$$

(6) $$\begin{array}{l} Accuracy=\frac{TP+TN}{TP+FN+TN+FP} \end{array}$$

(7) $$\begin{array}{l} Specificity=\frac{TN}{TN+FP} \end{array}$$

(8) $$\begin{array}{l} F\text{\_}Score=\frac{2\times Precision\times Recall}{Precision+Recall} \end{array}$$

Another popular measurement in machine learning is receiver operating characteristic (ROC), a probability curve; it is often plotted with true positive rate (TPR) on the y-axis and the false positive rate (FPR) on the x-axis. AUC, area under the curve, represents the degree or measure of separability. It tells how much the model is capable of distinguishing between classes. The higher the AUC, the better the model is at distinguishing between patients with the disease and no disease. For example, if AUC is 0.7, it means there is a 70% chance that the model will be able to distinguish between impairment and normal.

**Table 6:** Confusion matrix.

**True**	**Predicted**	
	0	1
0	TN	FP
1	FN	TP

## 3 Results

**Figure 1:**
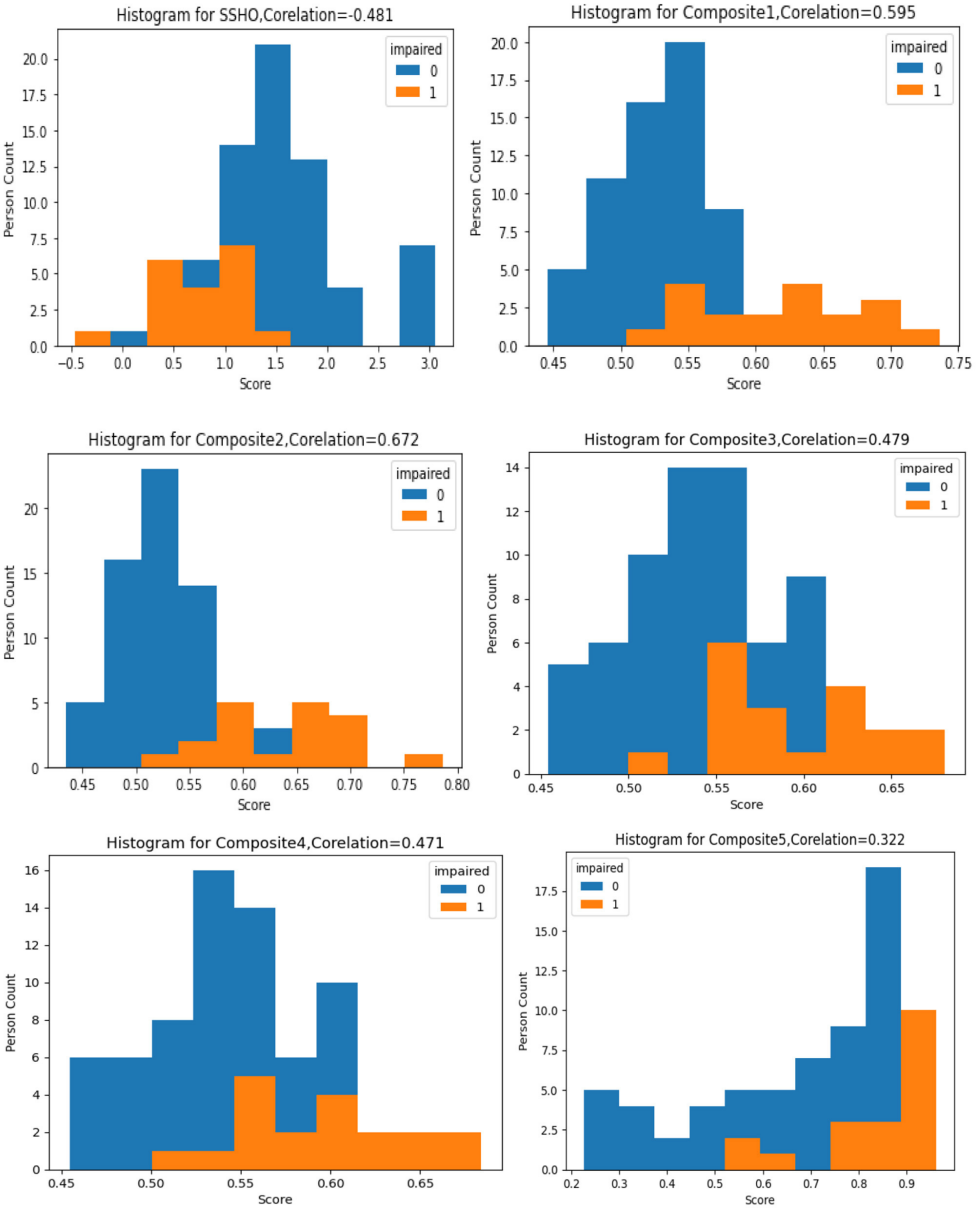
Histogram of six composite scores.

The figure labeled “Figure 1” displays a histogram with colored bars and labels indicating the impairment status shown by the data. Each histogram represents the distribution one of six composite scores: SSHO, Composite1, Composite2, Composite3, Composite4, and Composite5. The histogram provides information on the frequency of each composite score, with the SSHO score being one of the six scores plotted. This type of graph is helpful for identifying patterns in the data, such as the spread of scores and any potential outliers or anomalies. The color blue is used to indicate data with a normal impairment status, while the color orange is used to indicate data with an impaired status. This can help researchers quickly identify any differences in the distribution of scores between impaired and non-impaired groups.

### 3.1 Unsupervised data analysis

**Figure 2:**
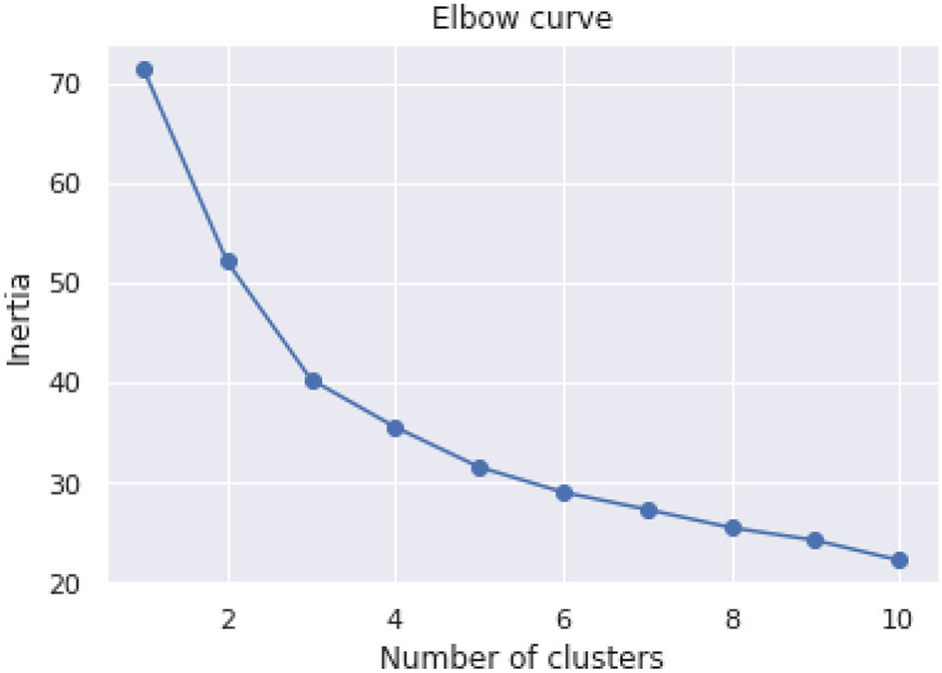
Elbow curve with 10 clusters.

The purpose of the cognitive assessment is to predict impairment status based on patients' assessment results without knowing what their true status are. Therefore, we would like to investigate the data to see if there are any patterns. Unsupervised data analysis was conducted on the dataset using the scikit-learn library's KMeans clustering algorithm. The algorithm was run with cluster numbers ranging from 1 to 10, and the sum of squared distances between each point and its nearest cluster center (known as inertia) was minimized. The results are plotted in Figure 2, where the number of clusters is shown against the Inertia measure. The plot suggests that the elbow point, where adding more clusters does not significantly improve the clustering, occurs at three or four clusters.

**Figure 3:**
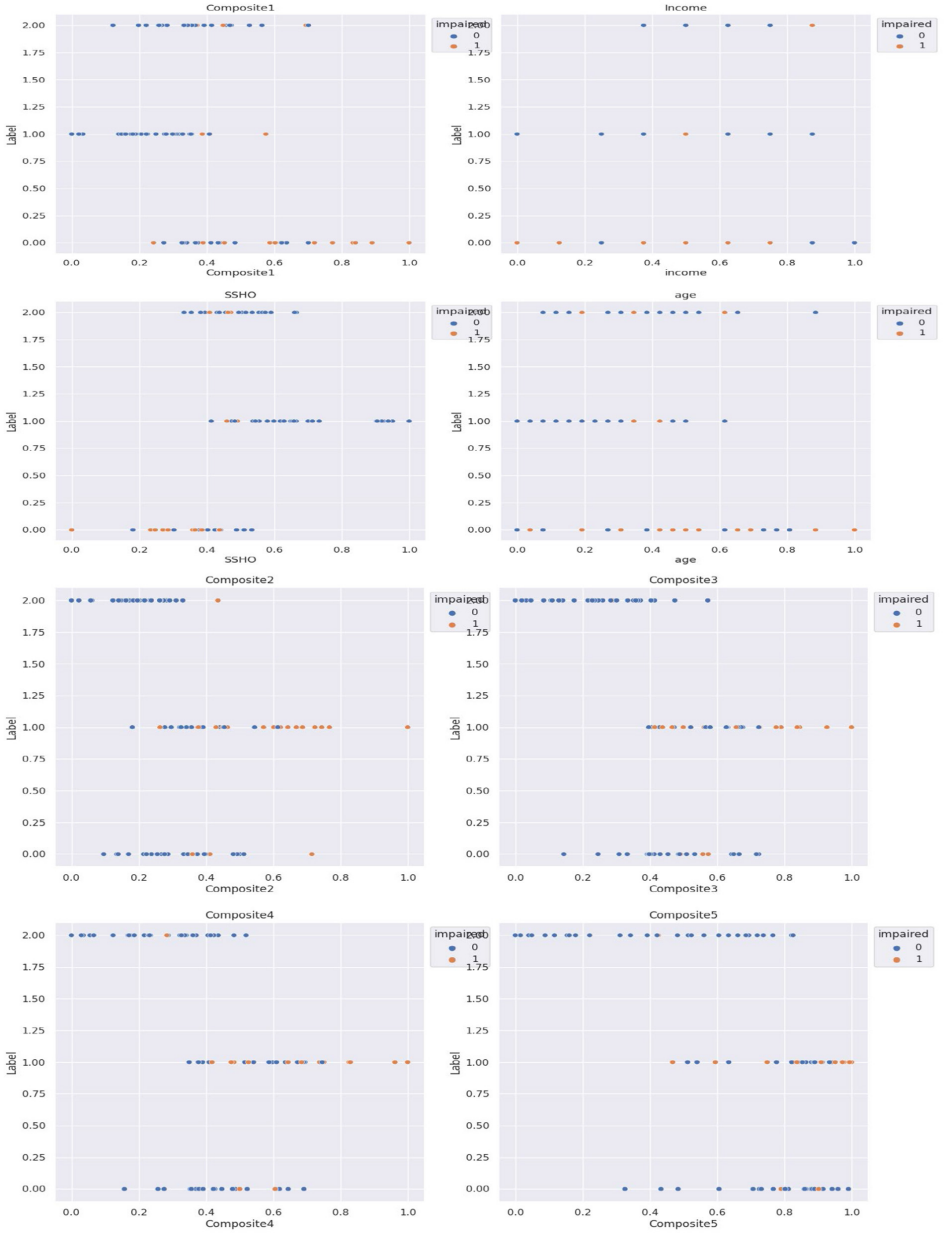
Five composite scores, SSHO, income, and age against the three cluster labels.

Subsequently, cluster analysis was performed with three clusters, and the patients were labeled as 0, 1, or 2 accordingly. Figure 3 shows the five composite scores (Composite1 to Composite5), SSHO, income, and age plotted against the cluster labels obtained, with the points colored according to their clinical impairment status.

The unsupervised cluster analysis with three clusters appears to reveal some patterns although the optimal number of clusters or the cutoff point for assigning labels is not entirely clear. Nonetheless, the analysis provides some insights into the grouping of patients based on their composite scores, SSHO, income, and age, and how they relate to their clinical impairment status. Further exploration and refinement of the clustering method may be necessary to gain a better understanding of the underlying structure of the data.

### 3.2 Supervised data analysis

To investigate the factors that affect the prediction of clinical impairment in this dataset, a supervised analysis was conducted using the clinical labels of impairment. By examining the relationships between the features and the labels, we can gain a better understanding of which variables are most relevant for predicting clinical impairment. This type of analysis can be useful for identifying potential risk factors or developing diagnostic tools to aid in early detection and intervention.

**Figure 4:**
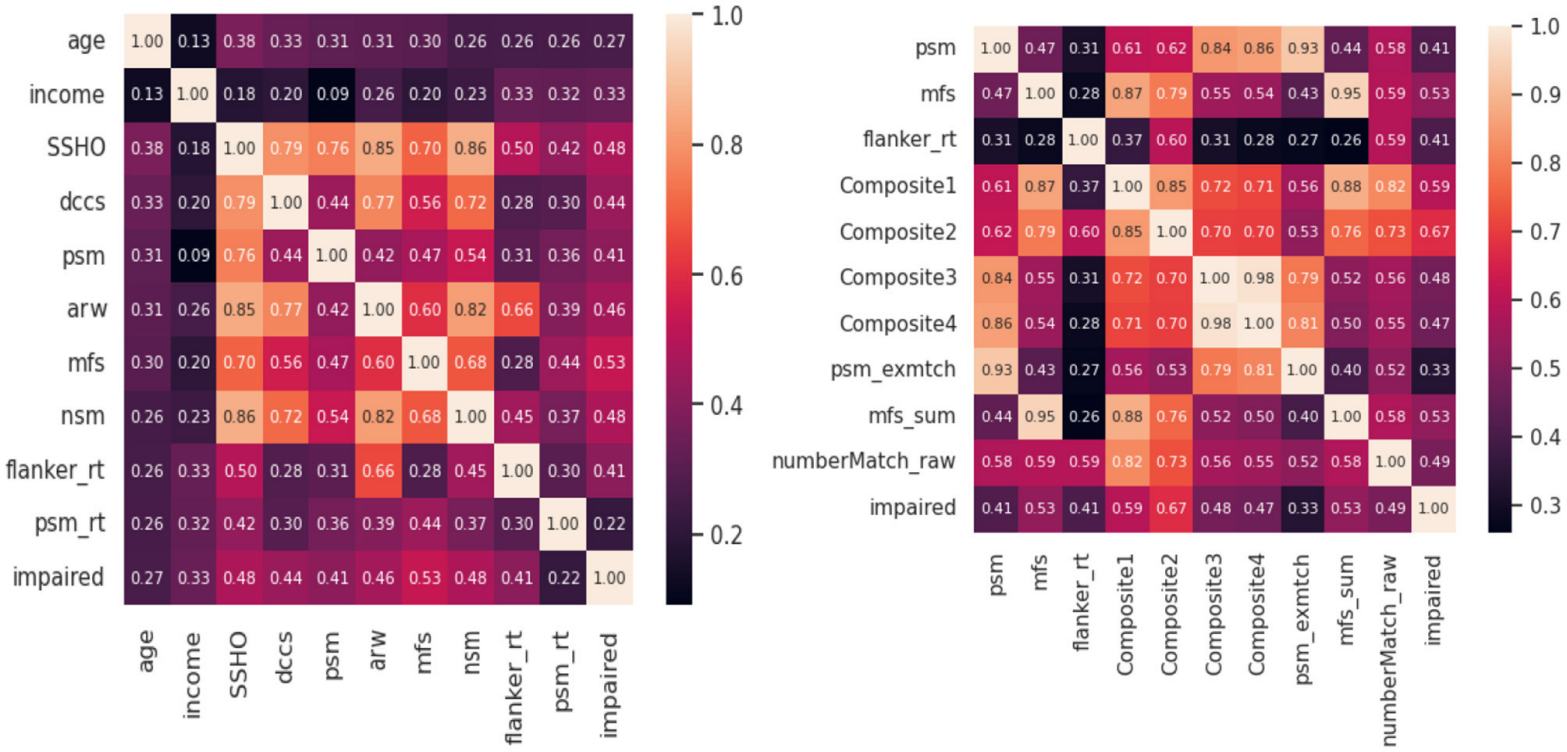
Heat map of 10 best features.

Feature analysis were conducted to check the best features among all the variables included in the model. Figure 4 shows two heat maps for the 10 best features. For the first heatmap, scores from HOIRT5D, demographic information, and response times were considered. It is observed that the order of impact of the features are the assessment scores MFS, NSM, SSHO, ARW, DCCS, PSM, and their response times, and demographic information income and age. The second heat map contains the five composite scores, and “Composite2” and “Composite1” are the two most important features.

Supervised machine learning was conducted with feature space listed in the design, and machine learning models were applied. For feature space that contains not only the assessment scores but also demographic information “race,” “gender,” “income,” “education,” and response times, such as F1, F2, F3, F4, F5, F6, and F7, some models such as GB and RF predicated all 86 patients correctly.

Although the performance a model should be examined by looking at their results for test data, results for all data are displayed in Table 7 for model RF for all 14 feature spaces. For feature space F1, F3, F5, and F6, all 86 patients were classified correctly.

**Table 7:** Results for all data from RF for 16 feature space.

**Feature**	**Precision**	**Recall**	**AUC**	***F*_measure_**	**Specificity**	**QWK**
F1	1	1	1	1	1	1
F2	1	1	1	1	1	1
F3	0.988	0.988	0.993	0.988	0.985	0.967
F4	0.977	0.977	0.966	0.977	0.985	0.932
F5	1	1	1	1	1	1
F6	0.988	0.988	0.993	0.988	0.985	0.967
F7	0.977	0.977	0.966	0.977	0.985	0.932
F8	0.965	0.965	0.959	0.965	0.97	0.901
F9	0.93	0.93	0.918	0.93	0.94	0.805
F10	0.953	0.953	0.951	0.953	0.955	0.87
F11	0.965	0.965	0.94	0.965	0.985	0.897
F12	0.942	0.942	0.925	0.942	0.955	0.834
F13	0.988	0.988	0.993	0.988	0.985	0.967
F14	0.965	0.965	0.921	0.965	1	0.893
F15	0.988	0.988	0.974	0.988	1	0.966
F16	0.965	0.965	0.94	0.965	0.985	0.897

**Figure 5:**
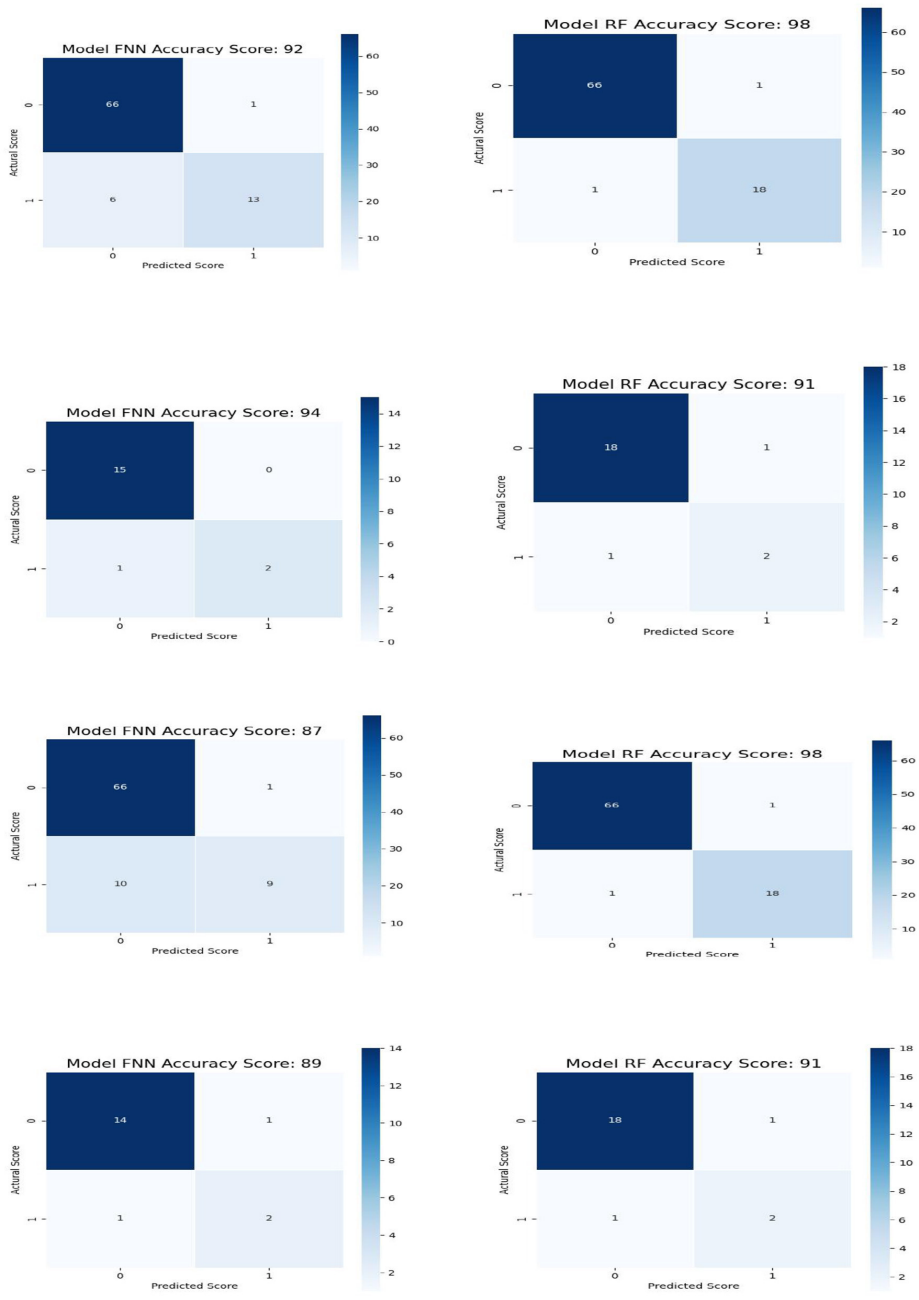
Confusion matrix for all the data and test data for FNN and RF for feature space F4 and GB and RF for feature space F7.

Figure 5 shows the confusion matrix for all the data (row-one) and test data(row-two) for FNN and RF for Feature Space F4. Row-three and row-four shows the confusion matrix for all the data and test data for FNN and RF for Feature Space F7. Feature space F4 used six assessment scores SSHO, dccs, psm, arw, mfs, nsm, derived from HOIRT-5D from the five cognitive assessment DCCS, PSM, ARW, MFS, and NSM. Feature space F7 used three assessment scores SSHO2D, dccs2D, psm2D, derived from HOIRT-2D from only two cognitive assessment DCCS and PSM. Compared with other feature space F1 and F5 that contains demographic information and response time, all 86 patients were correctly classified with RF; there was one case misclassified for F4 and two cases misclassified for F7.

**Figure 6:**
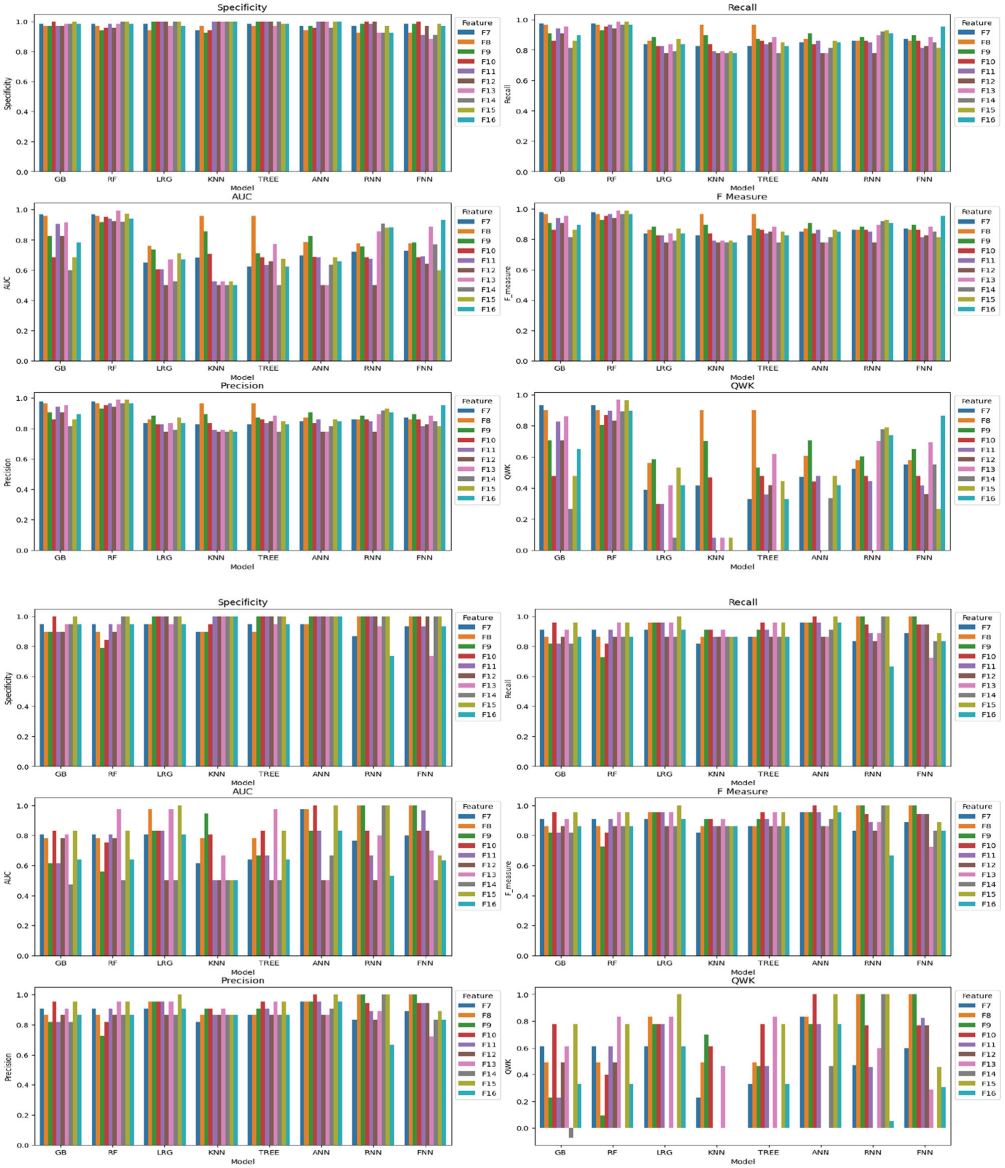
Specificity, recall, AUC, F, precision, and QWK for all models for all data (first three-row) and test data (last three-row) for feature space F7-F16.

In the analysis of Feature Space F7-F16, the performance of models with two cognitive assessments (DCCS and PSM) and composite scores was the primary focus. Figure 6 displays specificity, recall, AUC, F-measure, precision, and QWK for all models using the entire dataset (first three rows) and the test dataset (last three rows) for this feature space. The figure provides a visual representation of the relative performance of the models.

For the F-measure, which considers both precision and recall, the best performance on the test data for the ANN model was observed for F3, F6, F10, and F15. F3 utilized scores from HOIRT-5D along with demographic information, F6 used scores from HOIRT-2D and demographic information, F10 employed Composite3 (including scores from HOIRT-2D and age), and F15 utilized SSHO-2D and age.

When considering the QWK measure, which accounts for the agreement between predicted and actual labels, the ANN model showed the best performance for the test data with F3, F6, F10, and F15. All of these feature spaces included age information.

Examining the F-measure for features F15 and F16 (which focus on shorter assessments containing DCCS and PSM), the ANN model exhibited the best performance among the models.

Notably, when utilizing only one content area assessment (DCCS or PSM), the recall value for the ANN model was considerably lower for F13 and F14 compared to other feature spaces that included age.

It's important to note that the dataset consisted of only 86 participants, and the performance may vary with different datasets. However, based on the analysis, the inclusion of age in the feature space was found to be beneficial for prediction.

### 3.3 Performance of composite scores

**Figure 7:**
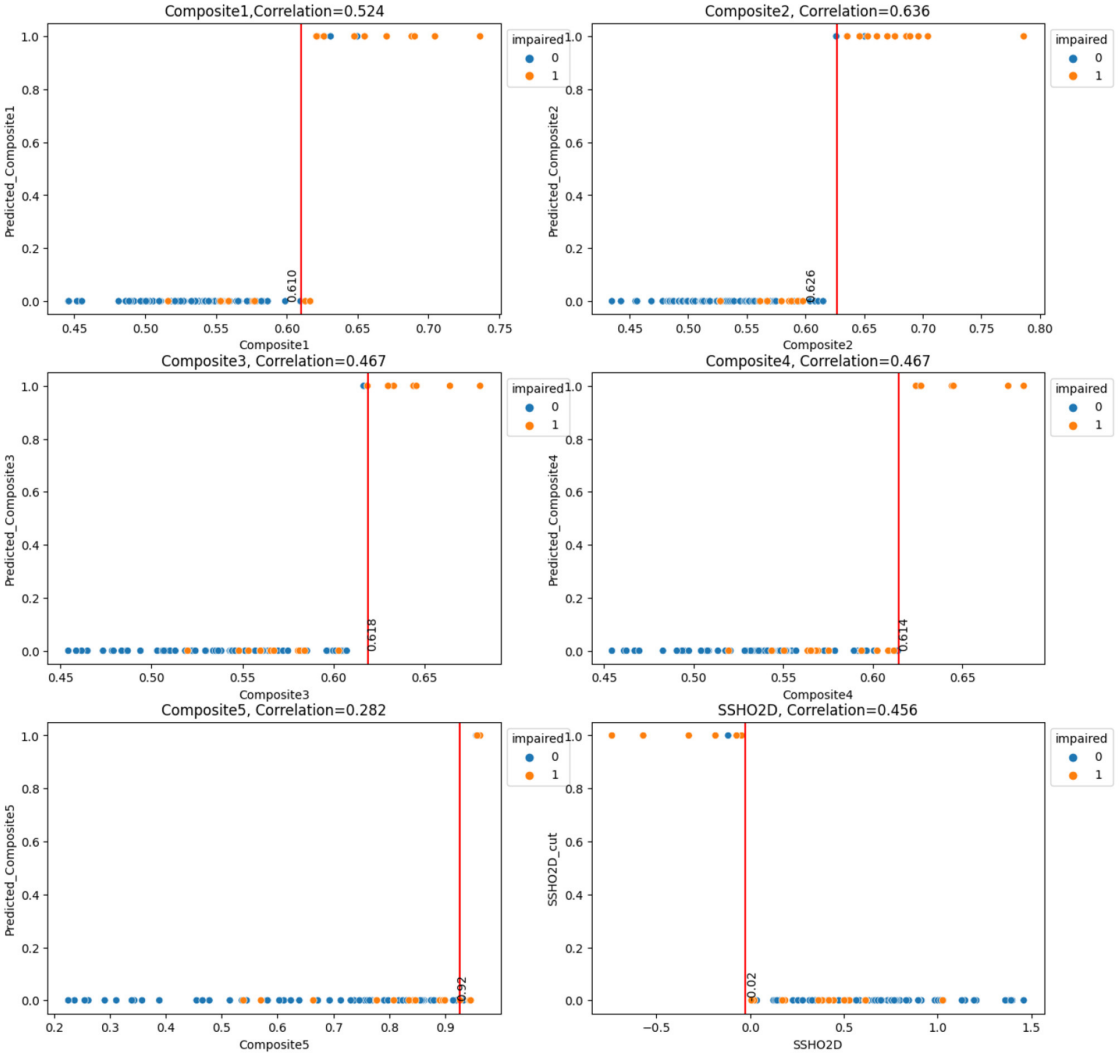
Scores against their predicted values(ANN) with Cut Points for the six Composite Scores for All 86 patients.

Next, we will examine the performance of the composite scores and how they performed using cut score and machine learning models. In general, ANN performed the best for most of the feature spaces, especially for composite scores as the only feature. Table 8 shows the result for test data from ANN for the 16 feature spaces. For test data, the feature space F2, F6, F10, and F15 performed perfectly. We checked the performance for all 86 patients from the ANN models and derived the best cut point and their performance for that cut point for all five composite scores. Figure 7 had six plots, and each shows the score values against their predicted impairment from the ANN model for all 86 patients for feature Composite1(F8), Composite2(F9), Composite3(F10), Composite4(F11), Composite5(F12), and SSHO2D, respectively. The best cut scores were plotted as the red vertical line, which was obtained through a computation searching with the minimum number of mismatched classifications. Table 9 shows the cut score and the number of mismatched cases for all 86 patients by the linear cut score for the five composite scores and SSHO2D. The table shows the misclassified number in machine learning for ANN for both test data and all 86 data and also results for all 86 patients for RF models.

**Table 8:** Results for the test data from ANN for 16 feature space.

**Feature**	**Precision**	**Recall**	**AUC**	***F*_measure_**	**Specificity**	**QWK**
F1	0.955	0.955	0.833	0.955	1	0.776
F2	0.955	0.955	0.833	0.955	1	0.776
F3	1	1	1	1	1	1
F4	0.955	0.955	0.974	0.955	0.947	0.831
F5	0.955	0.955	0.833	0.955	1	0.776
F6	1	1	1	1	1	1
F7	0.955	0.955	0.974	0.955	0.947	0.831
F8	0.955	0.955	0.974	0.955	0.947	0.831
F9	0.955	0.955	0.833	0.955	1	0.776
F10	1	1	1	1	1	1
F11	0.955	0.955	0.833	0.955	1	0.776
F12	0.864	0.864	0.5	0.864	1	0
F13	0.864	0.864	0.5	0.864	1	0
F14	0.909	0.909	0.667	0.909	1	0.463
F15	1	1	1	1	1	1
F16	0.955	0.955	0.833	0.955	1	0.776

**Table 9:** Number of misclassified patients for both machine learning model and linear cut for the six composite scores.

**Feature**	**Liner cut**		**Machine learning ANN**		**Machine learning (best)**
	**Point**	**Number of mismatch**	**All data**	**Test data**	**All data**
F8	0.61	11	11	1	3(RF)
F9	0.626	9	8	1	6(RF)
F10	0.618	12	14	0	4(RF)
F11	0.614	14	12	1	3(RF)
F12	0.925	14	17	1	5(RF)
SSHO2D	-0.026	14	12	0	1(RF)

Tables 10, 11 lists the 86 patients with their assessment scores, ethnic information, clinical diagnosis information, and their predicted labels from model RF F10 (Composite3), SSHO2D with linear cut, Composte5 with linear cut, and RF F16 with features containing dccs2D, psm2D, age, and their response times.

**Table 10:** Results for all patients.

	**Composite3**		**SSHO2D**		**Composite5**		**F16**	**Age**	**Gender**	**Income**	**Edu**	**Race**
**Impaired**	**Score**	**RF**	**Score**	**Linear**	**Score**	**Linear**						
		**Pd**		**Pd**		**Pd**						
0	0.597	0	0.229	0	0.92	0	0	77	F	8	9	W
0	0.513	0	0.899	0	0.639	0	0	78	F	9	11	W
0	0.564	0	0.473	0	0.791	0	0	80	M	7	8	W
0	0.562	0	0.51	0	0.611	0	0	75	F	9	8	W
0	0.546	0	0.703	0	0.798	0	0	75	F	4	6	W
0	0.507	0	0.897	0	0.358	0	0	67	F	5	8	W
0	0.618	0	-0.117	1	0.862	0	0	75	F	6	9	A
0	0.561	0	0.776	0	0.882	0	0	88	F	7	*	B
0	0.519	0	0.567	0	0.834	0	0	67	F	8	6	W
0	0.557	0	0.523	0	0.582	0	0	79	M	9	11	W
0	0.56	0	0.769	0	0.831	0	0	86	F	8	8	W
0	0.53	0	0.698	0	0.765	0	0	71	M	9	9	W
0	0.606	0	0.027	0	0.898	0	0	77	F	6	8	W
0	0.487	0	1.017	0	0.466	0	0	70	M	8	8	W
0	0.597	0	0.14	0	0.875	0	0	79	F	8	9	W
0	0.585	0	0.157	0	0.624	0	0	74	F	8	4	W
0	0.596	0	0.036	0	0.855	0	0	69	F	9	9	B
0	0.605	0	0.125	0	0.86	0	1	84	M	9	9	W
0	0.618	0	0.125	0	0.92	0	0	83	M	9	8	W
0	0.551	0	0.347	0	0.843	0	0	68	F	6	7	W
0	0.572	0	0.377	0	0.603	0	0	74	F	9	9	W
0	0.604	0	0.256	0	0.88	0	0	87	F	D	9	W
0	0.6	0	0.281	0	0.933	1	0	81	M	8	9	W
0	0.575	0	0.331	0	0.859	0	0	77	M	8	9	W
0	0.617	0	0.135	0	0.956	1	0	90	M	8	11	W
0	0.601	0	0.144	0	0.9	0	0	78	M	7	6	W
0	0.607	0	0.017	0	0.914	0	0	75	F	9	9	W
0	0.583	0	0.415	0	0.856	0	0	83	F	4	8	B
1	0.56	1	0.386	0	0.843	0	1	68	F	8	8	A
1	0.581	1	0.365	0	0.891	0	1	83	M	9	10	W
1	0.553	1	0.612	0	0.57	0	1	78	F	6	4	W
1	0.566	1	0.53	0	0.808	0	1	76	M	7	9	W
1	0.581	1	0.446	0	0.835	0	1	86	F	2	8	W
1	0.52	1	1.028	0	0.539	0	1	76	F	9	10	A
1	0.56	1	0.617	0	0.776	0	1	85	F	8	9	W
1	0.548	1	0.421	0	0.664	0	1	67	F	3	7	B
1	0.681	1	-0.737	1	0.958	1	1	80	F	6	6	W
1	0.644	1	0.022	0	0.963	1	0	93	F	5	4	W
1	0.619	0	-0.045	1	0.957	1	0	84	F	6	8	W
1	0.567	1	0.503	0	0.896	0	1	78	F	6	6	B
1	0.63	1	0.007	0	0.943	1	1	90	F	3	8	W
1	0.603	1	0.187	0	0.778	0	1	79	F	8	8	W
1	0.584	1	0.173	0	0.9	0	1	72	M	9	8	W
1	0.646	1	-0.327	1	0.847	0	1	81	F	5	4	W
1	0.633	1	-0.072	1	0.944	1	1	84	F	5	8	B
1	0.664	1	-0.57	1	0.946	1	1	75	M	7	4	W
1	0.63	1	-0.185	1	0.927	1	1	72	M	3	6	W

*indicate the value is missing.

**Table 11:** Results for all patients.

	**Composite3**		**SSHO2D**		**Composite5**		**F16**	**Age**	**Gender**	**Income**	**Edu**	**Race**
**Impaired**	**Score**	**RF**	**Score**	**near**	**Score**	**Linear**						
		**Pd**		**Pd**		**Pd**						
0	0.522	1	0.734	0	0.756	0	0	70	F	8	9	B
0	0.478	0	1.382	0	0.344	0	0	79	F	9	9	W
0	0.539	0	0.799	0	0.693	0	0	70	F	8	9	W
0	0.534	0	0.667	0	0.737	0	0	68	F	8	9	B
0	0.543	0	0.751	0	0.761	0	0	77	M	6	10	W
0	0.546	0	0.679	0	0.731	0	0	80	F	7	8	W
0	0.57	1	0.632	0	0.874	0	0	80	M	9	9	B
0	0.51	0	0.907	0	0.545	0	0	70	M	9	9	W
0	0.546	0	0.659	0	0.79	0	0	74	F	8	8	W
0	0.53	0	0.847	0	0.713	0	0	72	F	9	8	W
0	0.454	0	1.399	0	0.262	0	0	71	F	7	9	W
0	0.51	0	0.914	0	0.605	0	0	75	F	6	9	W
0	0.548	1	0.668	0	0.831	0	0	73	F	7	11	W
0	0.552	0	0.527	0	0.882	0	0	75	M	9	9	W
0	0.473	0	1.362	0	0.311	0	0	76	F	8	9	W
0	0.544	0	1.017	0	0.693	0	0	83	F	2	6	W
0	0.508	0	1.029	0	0.515	0	0	74	F	9	9	W
0	0.465	0	1.393	0	0.291	0	0	74	F	5	8	W
0	0.584	0	0.317	0	0.581	0	0	83	F	9	9	W
0	0.503	0	1.03	0	0.339	0	0	78	F	9	8	W
0	0.538	0	0.696	0	0.455	0	0	75	F	4	6	W
0	0.462	0	1.207	0	0.255	0	0	70	F	7	6	W
0	0.53	0	0.617	0	0.747	0	0	69	M	5	6	W
0	0.535	0	0.756	0	0.769	0	0	78	F	9	9	W
0	0.486	0	1.195	0	0.389	0	0	70	F	8	9	W
0	0.537	0	0.833	0	0.769	0	0	78	F	8	10	W
0	0.51	0	0.784	0	0.672	0	0	68	F	7	9	B
0	0.523	0	0.682	0	0.536	0	0	69	F	8	8	W
0	0.544	0	0.563	0	0.817	0	0	71	M	9	9	W
0	0.484	0	1.147	0	0.237	0	0	68	F	7	8	W
0	0.494	0	1.015	0	0.478	0	0	71	F	9	9	B
0	0.546	0	0.564	0	0.824	0	0	72	M	7	*	W
0	0.565	0	0.616	0	0.863	0	0	78	M	9	10	W
0	0.544	0	0.477	0	0.876	0	0	74	M	6	8	A
0	0.459	0	1.46	0	0.226	0	0	67	F	9	11	W
0	0.506	0	0.986	0	0.581	0	0	73	F	5	9	W
0	0.524	0	1.002	0	0.672	0	0	81	M	5	9	U
0	0.479	0	1.13	0	0.389	0	0	70	F	8	7	W
0	0.548	0	0.584	0	0.879	0	0	81	M	9	11	W

*indicate the value is missing.

It is clear that linear cuts for predicting impairment is fall compared to machine learning models. The misclassification rate for all linear cut for the composite scores are higher than those from machine learning models. For ANN with feature space F15 that contains SSHO2D and age, the prediction is perfect.

### 3.4 Bootstrap and replications

**Figure 8:**
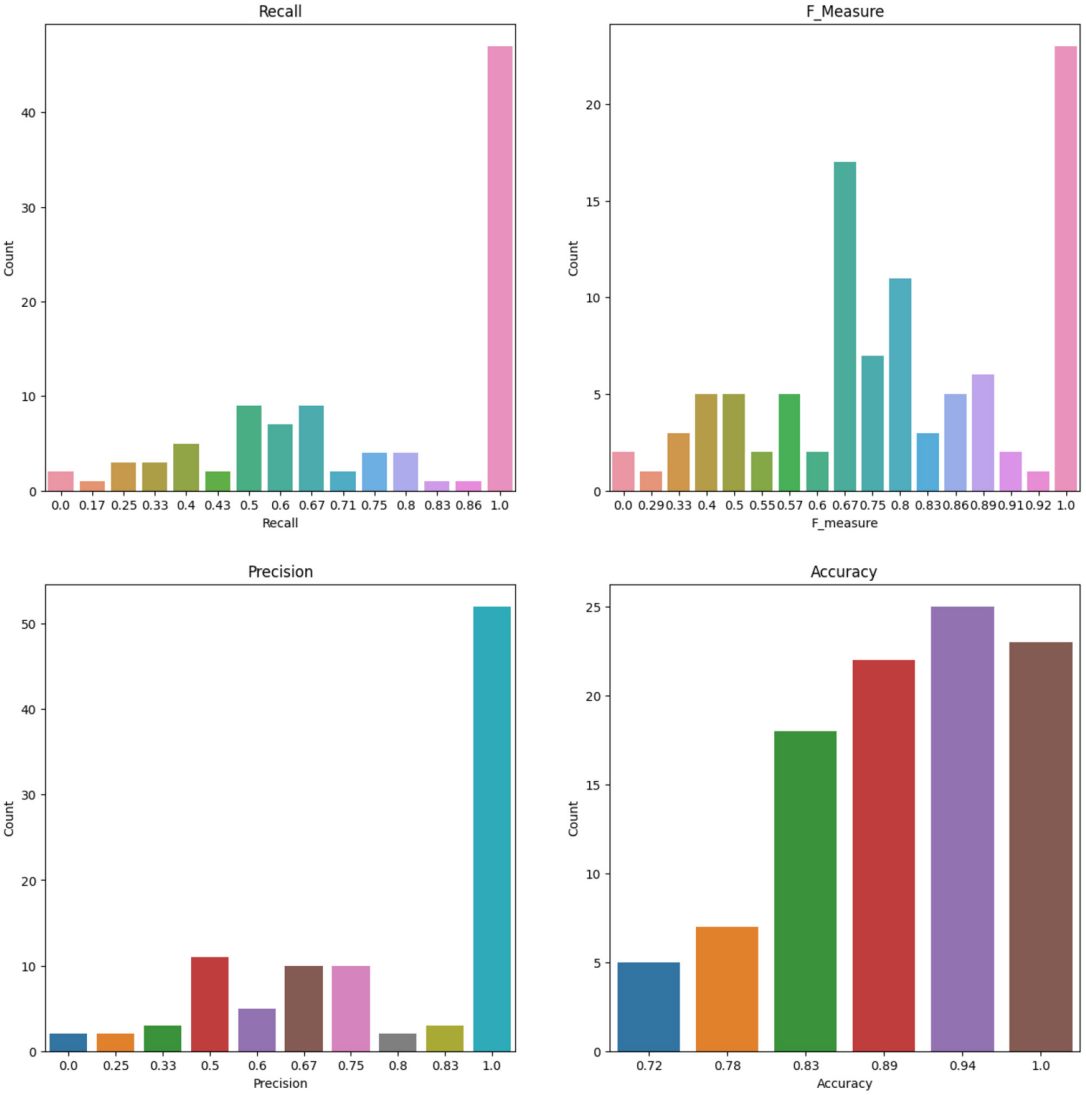
The Number of Count for Recall, F measure, Precision and Accuracy from 100 Bootstrap for RF model for Features containing SSHO2D and age.

**Figure 9:**
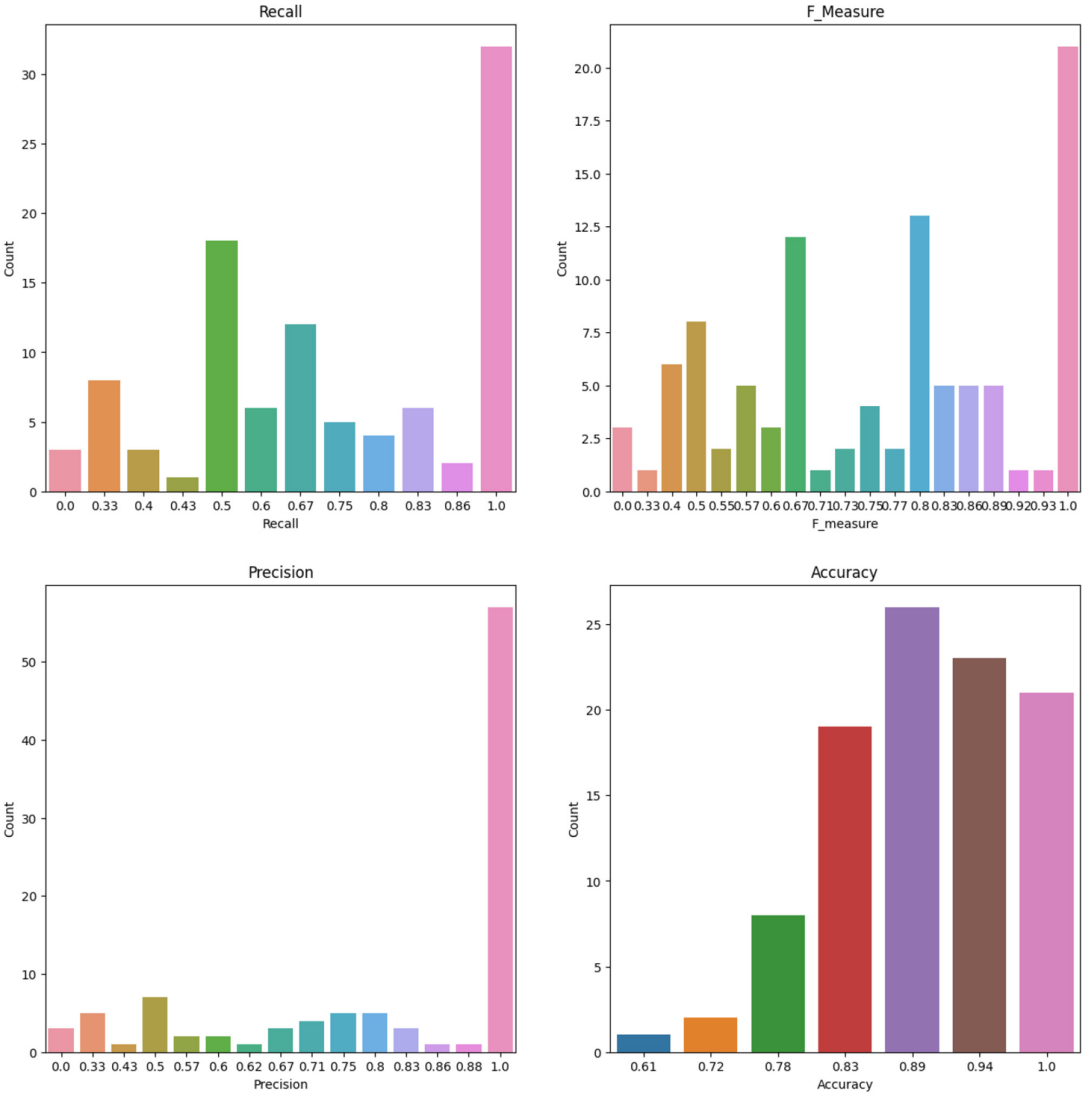
The Number of Count for Recall, F measure, Precision and Accuracy from 100 Bootstrap for ANN model for Features containing dccs2D, psm2D, dccs-rt, psm-rt, and age.

To account for the potential instability of the results due to a small sample size, the original dataset of 86 patients underwent 100 rounds of bootstrapping, where the same sample size was maintained and the training process was repeated each time. Means and standard deviations were calculated from these replication processes, and the results are displayed in Table 12 for the RF and ANN models for some selected features. Figure 8 shows the number of count for precision, recall, accuracy, F-measure, and specificity from 100 Bootstrap for RF model for features containing {*age, SSHO*2*D*} with their average values of 0.803, 0.758, 0.902, 0.742, and 0.951, respectively. Figure 9 shows the number of count for precision, recall, accuracy, F-measure, and specificity from 100 Bootstrap for ANN model for features containing {*age, dccs*2*D, psm*2*D, dccs*−*rt, psm*−*rt*} with their average values of 0.824, 0.706, 0.899, 0.733, and 0.955, respectively.

**Table 12:** The means and standard deviations of 100 replications for test data for RF and ANN with feature space F15 and F16.

	***RF***				***ANN***			
**Measure**	{***SSHO***2***D**, **age***}		{***dccs***2***D**, **psm***2***D***}		{***SSHO***2***D**, **age***}		{***dccs***2***D**, **psm***2***D***}	
			{***age**, **rt***}				{***age**, **rt***}	
	**Mean**	**SD**	**Mean**	**SD**	**Mean**	**SD**	**Mean**	**SD**
Precision	0.803	0.246	0.794	0.295	0.83	0.226	0.824	0.253
Recall	0.758	0.272	0.671	0.313	0.718	0.262	0.706	0.261
Accuracy	0.902	0.079	0.903	0.07	0.9	0.077	0.899	0.078
Fmeasure	0.742	0.223	0.694	0.269	0.738	0.212	0.733	0.226
Specificity	0.951	0.067	0.961	0.057	0.956	0.056	0.955	0.065

For the ANN model, which was trained on a feature set consisting of SSHO2D, age, income, race, 30-DCCSrt, and psm-rt, a total of 20 rounds of bootstrap resampling were performed on the dataset. Precision, recall, accuracy, F1, and specificity were computed for the model and had values of 0.833, 0.748, 0.944, 0.777, and 0.983, respectively. The results provide evidence that including item-level response times and demographic information as features improved the model's prediction performance, particularly with regard to recall.

The study also assessed the impact of reducing the number of content areas assessed on the model's performance. When the feature space contained demographic information and the total item response times, the recall value reduced from 0.772 to 0.728 when moving from five content areas to two content areas. Similarly, when the feature space contained demographic information and the overall score from HOIRT, the recall value reduced from 0.762 to 0.674 when moving from five content areas to two content areas.

**Figure 10:**
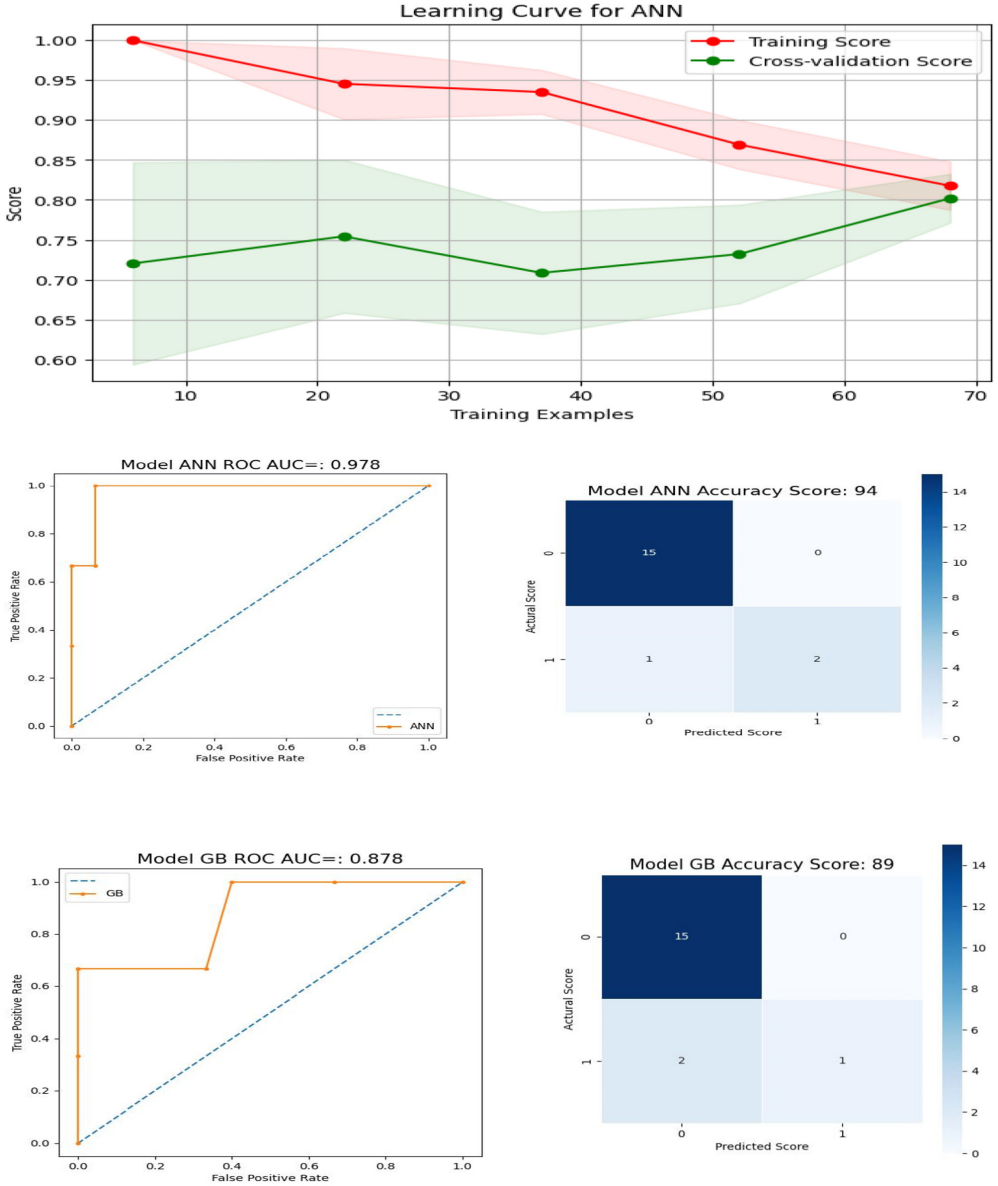
Learning Curve, ROC Curve and Confusion Matrix for ANN and GB for features containing age, number of correct scores, and their total response times for DCCS and PSM.

To facilitate the seamless integration of the machine learning scoring model into the system and due to the challenges associated with collecting demographic information such as gender, education, income, or race, our last model were designed to focus on features that include age, the number of correct scores for PSM and DCCS, as well as their total response times: “*age*,″“*psm*,″“*DCCS*,″“*dccs*_*rt*,″ and “*psm*_*rt*″. This approach was chosen for the sake of simplicity and practicality, aiming to ensure a smooth implementation of the machine learning scoring model while mitigating the complexities associated with collecting demographic data. The results for ANN and GB, along with the AUC (area under the curve) and confusion metrics, are visually presented in Figure 10. The top row of the plot displays the learning curve for the optimal ANN model with specific configurations: *solver* =′*sgd*′, *activation* =′*tanh*′, *alpha* = 0.01, *hidden*_*layer*_*sizes* = (150, 100, 50), and *max*_*iter* = 1000. Notably, the training and validation scores closely converge, suggesting the model's effectiveness (24).

## 4 Discussion

The current study utilized clinical data from 86 patients who completed five cognitive assessments. Unsupervised cluster analysis was performed to identify patterns and classify patients based on features. Composite scores were computed using both scores from higher order two-parameter item response theory (HOIRT) models and classical number of correct score models. Five possible linear combinations of scores were derived to investigate the potential application of composite scores in predicting impairment.

Supervised machine learning was employed to predict impairment for the 86 patients using various machine learning models and feature spaces. RF and GB tended to perform the best for feature spaces F1-F4, which contained more features such as the six assessment scores, “race,” “gender,” “income,” “education”, and response times. All 86 patients were correctly classified using these machine learning models with those feature spaces.

The optimal cut point for each of the six composite scores was determined by minimizing the number of mismatched patients out of the 86 patients. The number of mismatched cases was 11, 9, 12, 14, 14, and 14 for Composite1, Composite2, Composite3, Composite4, Composite5, and SSHO2D, respectively. Among the composite scores, Composite2, which was derived from six HOIRT-5D scores, response times, and age, had the smallest number of mismatched cases. When using only SSHO2D or Composite4 and applying a cut point of -0.026 for SSHO2D and 0.925 for Composite4, there were 14 mismatched cases. However, when running the RF model using these scores as the only feature, the number of mismatched cases reduced to 4. This suggests that using composite scores as the sole predictor in a machine learning model can improve the accuracy of cognitive impairment prediction compared to using individual domain scores or composite scores with cut points.

When only composite scores were used as features in the feature space, the RF model produced a significantly smaller number of mismatched cases compared to using cut points. This finding suggests that composite scores may serve as a useful predictor of impairment and that machine learning models such as RF can be a valuable tool for predicting impairment in clinical settings.

Additionally, the study conducted a comparison of prediction performance between two groups: patients who underwent five assessments (DCCS, PSM, ARW, MFS, and NSM) and those who only took two assessments (PSM and DCCS). The findings indicated that the model achieved better performance when more assessments were taken. However, for certain models, utilizing two assessments (DCCS and PSM) and predicting using the assessment scores along with age information yielded comparable results. When comparing composite scores derived from five vs. two assessments, the results showed that using only two assessments (DCCS and PSM) and adding demographic information to the three assessment scores (SSHO2D, dccs2D, and psm2D), the RF model was able to correctly predict all 86 patients. However, when using feature space F7, which only contains the three scores and does not include demographic information, the RF model had one “normal” patient classified as “CI” (cognitive impairment) and one “CI” patient classified as “normal”. Therefore, including demographic information such as age is important in cognitive impairment prediction models to improve accuracy.

Given the small training size, the stability of the results becomes a concern. To mitigate this issue, bootstrap techniques were employed on specific feature spaces. The training results were computed based on 100 bootstrap samplings, allowing for the calculation of mean and standard deviations. Machine learning model RF with features that contains composite score and age yielded precision, recall, accuracy, F1, and specificity of values 0.803, 0.758, 0.902, 0.742, and 0.951, respectively. The composite score was derived from a 2-parameter higher order item response theory (HOIRT) model with two assessments DCCS and PSM.

The final model takes into account the inherent challenges of gathering demographic information, including gender, education, income, and race. It is intentionally crafted to concentrate on a select set of features, specifically age, raw response data, and response times, with a specific focus on the DCCS and PSM content areas. The graphical representations of the AUC and confusion matrices for the ANN and GB models provide evidence of the model's commendable performance.

In summary, the study emphasizes the significance of incorporating demographic information and item-level response times into the feature space for machine learning models to achieve improved prediction performance (25, 26). It suggests that relying on a single, simple cut point for a composite score, regardless of how well it is derived, may not yield optimal outcomes. Instead, employing machine learning models that utilize scores derived from HOIRT2D and encompass features such as age can lead to effective prediction models.

In order to improve the performance and generalizability of machine learning models, a larger training size is often necessary. To utilize the model in practice, it is important to collect a representative and diverse dataset for training the models. This helps to ensure that the models learn from a wide range of samples and can generalize well to unseen data.

For forthcoming research endeavors, our objective is to integrate the temporal aspects of patients' PSM tests such as action sequences of patients taking the test and survey responses into machine learning models, with the aim of detecting abnormal behaviors and identifying patients with Alzheimer's disease/cognitive impairment (AD/CI). Notably, in the field of literature review, significant advancements have been made in the development and exploration of novel machine learning models.

Jiao and colleagues (27) offered an insightful review of the literature in this context with emphasis in measurement and assessments. Furthermore, in a recent investigation conducted by Ke and colleagues (28), they explored the application of a deep convolutional neural network (CNN) and a frequency channel-based CNN (FCCNN) for the precise and expedited identification of depression. The FCCNN operates in the frequency domain, allowing it to capture patterns and features that may not be readily discernible in the spatial domain. This approach draws parallels with term document weighting (TDW) in natural language processing [NLP, (29)], where attention is paid to term frequencies to discover significant features. The study's findings indicated that FCCNN achieved a high classification accuracy when applied to a publicly available EEG dataset related to major depressive disorder. These innovative approaches showcase the ongoing progress in the field and hold promise for future research.

Functional magnetic resonance imaging (fMRI) provides a comprehensive insight into brain activity and has emerged as a pivotal tool in the identification of attention-deficit/hyperactivity disorder (ADHD), a prevalent behavioral disorder in children. Researchers have harnessed the capabilities of fMRI in conjunction with various neural measures, including electroencephalography (EEG), to investigate a wide spectrum of cognitive processes, encompassing perception, memory, decision-making, and emotional responses (30). Additionally, the combination of a patient's clinical status with perfusion-CT imaging, as highlighted by Strambo and colleagues (31), holds considerable promise. The integration of fMRI data with our cognitive assessment scores, such as those derived from the DCCS and PSM, in conjunction with demographic information is considered powerful in the detection and comprehension of AD/CI. Our commitment to advancing this aspect of our study underscores our dedication to enhancing our understanding of cognitive function and its potential variations across diverse individuals and populations.

Our ultimate objective is to develop the MyCog system, which can autonomously provide accurate and efficient predictions of patients' AD/CI. The continuous updating of hyperparameters with newly acquired data is of paramount importance in achieving this goal. In their research, Ke and colleagues (30) introduced a dual-CNN (convolutional neural network) methodology to enhance brain e-health service platforms. This innovative system leverages automatic machine learning techniques to construct a dual-CNN model that excels in both accuracy and efficiency. Furthermore, it empowers a deep Neural network (DNN)-based model to continually enhance its own performance through the optimization of hyperparameters and adaptation to incoming data. Their study showcases the potential of machine learning and hyperparameter optimization in the development of a robust and adaptive system, bringing us closer to the realization of the MyCog system's goal of providing accurate and efficient predictions for AD/CI patients.

## Data availability statement

The raw data supporting the conclusions of this article will be made available by the authors, without undue reservation.

## Ethics statement

The studies involving humans were approved by the Institutional Review Board of Northwestern University. The studies were conducted in accordance with the local legislation and institutional requirements. The participants provided their written informed consent to participate in this study.

## Author contributions

LY: Conceptualization, Formal analysis, Methodology, Software, Writing—original draft, Writing—review & editing. AK: Conceptualization, Data curation, Funding acquisition, Writing—review & editing. CN: Funding acquisition, Project administration, Writing—review & editing. EH: Data curation, Software, Writing—review & editing. MW: Funding acquisition, Writing—review & editing. RG: Funding acquisition, Writing—review & editing. YS: Formal analysis, Writing—review & editing. ED: Formal analysis, Writing—review & editing. SC: Analysis, Writing—review & editing. RL: Funding acquisition, Review & editing. LC: Writing & editing. JB: Sample collection, Writing & editing.

## Footnotes

^1^Total response times for each of the three tests.

^2^Total response times for psm, dccs, and arw.

^3^relu(x)=max(0,x), *tanh* = (*e*^*x*^−*e*^−*x*^)/(*e*^*x*^+*e*^−*x*^).
